# Dengue Virus Serotype 4 Is Responsible for the Outbreak of Dengue in East Java City of Jember, Indonesia

**DOI:** 10.3390/v12090913

**Published:** 2020-08-20

**Authors:** Aryati Aryati, Billy J. Wrahatnala, Benediktus Yohan, May Fanny, Faradila K. N. Hakim, Eka Putri Sunari, Nelly Zuroidah, Puspa Wardhani, Marsha S. Santoso, Dominicus Husada, Ali Rohman, Siti Nadia Tarmizi, Justus T. O. Sievers, R. Tedjo Sasmono

**Affiliations:** 1 Department of Clinical Pathology, Faculty of Medicine, Universitas Airlangga, Surabaya 60286, Indonesia; dr_aryati@yahoo.com (A.A.); puspa_pk@yahoo.co.id (P.W.); 2 Institute of Tropical Diseases, Universitas Airlangga, Surabaya 60115, Indonesia; 3 Master Program of Tropical Medicine, Faculty of Medicine, Universitas Airlangga, Surabaya 60286, Indonesia; drbillyjordanw@gmail.com (B.J.W.); fara.faradilahakim@gmail.com (F.K.N.H.); 4 Eijkman Institute for Molecular Biology, Jakarta 10430, Indonesia; yohan@eijkman.go.id (B.Y.); marshasantoso@eijkman.go.id (M.S.S.); jto.sievers@gmail.com (J.T.O.S.); 5 Clinical Pathology Sub-Specialization Program, Universitas Airlangga, Surabaya 60286, Indonesia; dr.mayfannytanzilia@gmail.com; 6 Department of Clinical Pathology, Faculty of Medicine, Universitas Ciputra, Surabaya 60219, Indonesia; 7 Clinical Pathology Specialization Program, Universitas Airlangga, Surabaya 60286, Indonesia; siputrisunari@yahoo.com (E.P.S.); nelly.zuroidah-2016@fk.unair.ac.id (N.Z.); 8 Department of Child Health, Faculty of Medicine, Universitas Airlangga, Surabaya 60286, Indonesia; dominicushusada@yahoo.com; 9 Department of Chemistry, Faculty of Science and Technology, Universitas Airlangga, Surabaya 60115, Indonesia; alirohman@fst.unair.ac.id; 10 Directorate of Vector-Borne and Zoonotic Disease, Ministry of Health of Indonesia, Jakarta 12950, Indonesia; nadiawiweko@gmail.com

**Keywords:** dengue, molecular epidemiology, serotype, Java, Indonesia

## Abstract

Outbreaks of dengue virus (DENV) in Indonesia have been mainly caused by the DENV serotype-1; -2; or -3. The DENV-4 was the least-reported serotype in Indonesia during the last five decades. We recently conducted a molecular epidemiology study of dengue in the Jember regency, East Java province, Indonesia. Dengue is endemic in the region and outbreaks occur annually. We investigated the clinical characteristics and etiology of dengue-like febrile illness in this regency to understand the disease dynamics. A total of 191 patients with clinical symptoms similar to dengue were recruited during an 11-month study in 2019–2020. Children accounted for the majority of cases and dengue burden was estimated in 41.4% of the cases based on NS1 antigen, viral RNA, and IgG/IgM antibody detection with the majority (73.4%) being primary infections. Secondary infection was significantly associated with a higher risk of severe dengue manifestation. All four DENV serotypes were detected in Jember. Strikingly, we observed the predominance of DENV-4, followed by DENV-3, DENV-1, and DENV-2. Genotype determination using Envelope gene sequence revealed the classification into Genotype I, Cosmopolitan Genotype, Genotype I, and Genotype II for DENV-1, -2, -3, and -4, respectively. The predominance of DENV-4 in Jember may be associated with a new wave of DENV infections and spread in a non-immune population lacking a herd-immunity to this particular serotype.

## 1. Introduction

The dengue virus (DENV) is a member of the *Flaviviridae* family that causes a systemic infection simply known as dengue. DENV maintains arboreal-human transmission cycles via the *Aedes* mosquito vector, with historical records of cases matching the symptoms of infection dating as far back as 265 AD in China [1]. DENV’s ~10.7 kb single-stranded positive-sense RNA genome encodes three structural (C, prM/M, E) and seven non-structural (NS1, NS2A, NS2B, NS3, NS4A, NS4B, NS5) proteins [2,3]. The four serotypes (DENV-1, -2, -3, and -4) are able to cause repeated and simultaneous infections with varying clinical manifestations, most commonly an acute febrile illness, accompanied by myalgia, arthralgia, headache, and a rash, in the case of classical dengue fever (DF) [4,5]. In more extreme cases, the infection can progress to dengue hemorrhagic fever (DHF), and the frequently fatal dengue shock syndrome (DSS), making dengue a significant public health problem in tropical and subtropical areas of the world, including Indonesia, driven by the global distribution of mosquito vectors, among other factors [4,5].

The serotypes of DENV are antigenically different, enabling repeated and simultaneous infections, as well as being genetically clearly distinct, with separate genotypes within each serotype, which demonstrate varying pathogenicity and infectivity [6,7,8]. Phylogenetic analyses of DENV genomes have recently also allowed an expanded understanding of the factors driving the virus’ evolution and pathogenicity, as well as its historical origins [8,9].

Indonesia has a long history of dengue, with metropolitan centers Jakarta and Surabaya reporting cases since 1968 [10]. Infections are widespread, with all provinces and 85.6% of cities in the country reporting at least one case of DHF in 2018 [11]. In the same year, East Java province recorded the second highest number of DHF cases (8449 in total) and the highest number of total fatalities (84 in total) in Indonesia, with an incidence rate of 21.4/100,000 [11]. However, while the history and development of DENV is well-characterized in the province’s capital Surabaya [12,13,14] and Madura [15], there is a distinct lack of studies elucidating the nature of dengue in the Jember regency, the third largest city in East Java after Surabaya and Malang.

The expansion of the monitoring of DENV evolution in less-studied areas such as Jember is essential in implementing effective public health strategies to mitigate and avoid future dengue epidemics. Such epidemics can occur when new genotypes of DENV emerge in or are introduced to naïve populations, as has been reported previously [16,17]. As DENV genotypes have been reported to show antigenic differences [18], new genotypes present a significant risk, even to populations with a history of exposure to other DENV strains.

The spread of a rare DENV serotype would have a similar potency as an epidemic. Previous analyses of dengue outbreaks in Indonesia have shown these to be overwhelmingly caused by DENV-1–3 [14,19,20], with cases of serotype shift over time [12,13]. While DENV-4 has circulated in East Asia and other parts of South-East Asia over the past 60 years [9], there have been no reports of outbreaks associated with DENV-4 in Indonesia, except for an isolated report from Jakarta based on a limited number of partial genomic sequences [21]. Due to this rarity of infections, DENV-4 is also the least studied of the serotypes, with five identified genotypes (I–V), one of which is exclusively sylvatic (IV), with genotype-II being split into -IIa and -IIb [9,18].

The process of monitoring dengue cases in Indonesia must also account for ambiguity in clinical symptoms and cross-reactive antibodies affecting the identification of patients. Infection with other *Aedes*-transmitted viruses often show very similar symptoms to those of dengue and will often share spatio-temporal distribution with dengue. One such disease, chikungunya, caused by the Alphavirus chikungunya virus (CHIKV), not only shares a large amount of clinical symptoms with DF, but is also widespread in Indonesia [22]. While point-of-care rapid antibody testing kits for DENV, such as those developed for IgG and IgM, can exclude chikungunya patients, these immunoglobulins may be cross-reactive for DENV if the patient is infected with another *Flaviviridae* virus, such as the Zika virus (ZIKV) [23] or the Japanese encephalitis virus (JEV) [24]. Zika, a member of the same *Flaviviridae* family as DENV, causes clinical symptoms which may easily be confused with DF and there is evidence to support the notion that it is widespread in Indonesia, with a large number of undiagnosed/misdiagnosed cases due to the low severity of symptoms [25].

In this study, we performed a molecular epidemiology study of DENV and other arboviruses circulating in Jember regency, East Java. Our major aims were to inspect both clinical epidemiology and virological aspects of dengue in Jember. Here, we highlight the role of DENV-4 in causing an outbreak in the region and describe the genetic and epidemiological make up that it may contribute.

## 2. Materials and Methods

### 2.1. Ethical Considerations for the Commencement of the Study

The study was initiated after obtaining ethical approval from the Research Ethics Committee of the Faculty of Medicine Universitas Airlangga, Surabaya, Indonesia (Ethical Approval No. 156/EC/KEPK/FKUA/2019, 17 May 2019). The voluntary involvement into the study was ensured by obtaining written informed consent from all patients and/or children’s legal guardians.

### 2.2. Study Design and Patient Recruitment

The study was designed as a cross-sectional study performed in the Jember regency, East Java Province, Indonesia. Jember is the third largest urban area in East Java after Surabaya and Malang, located at −8°10′8″ S latitude, 113°42′8″ E longitude with an area of 3306 km^2^ and an average altitude of 0–500 m above sea level (masl). The population consists of 2,450,668 inhabitants with 0.41% population growth in 2019, living in 31 districts and 248 villages [26]. Jember is located approximately 200 km to the southeast from Surabaya, the capital city of East Java.

Study subjects’ recruitment was conducted for 11 months from May 2019 to March 2020, covering a monsoon season occurring typically from November to April, peaking in January, which is also the dengue peak season in the area. Patients suspected of having dengue were recruited at six hospitals/healthcare centers, namely RS Jember Klinik, RSD Dr. Soebandi, RS Citra Husada, Klinik Dr. M. Suherman, Klinik Dokterku Taman Gading, and Puskesmas Sumbersari, Jember. The inclusion criteria were febrile patients willing to participate in the study, aged up to 80 years old with a body temperature of ≥38 °C for less than 5 days of fever, accompanied by at least two of the clinical symptoms of dengue, i.e., headache, retro-orbital pain, myalgia, arthralgia/bone pain, and rash, as described in the World Health Organization, South-East Asia Regional Office (WHO-SEARO) 2011 guidelines [5]. The exclusion criteria were patients with fever and upper respiratory tract infections and/or those diagnosed as non-dengue. An interview session was performed by a doctor or research nurse to record demographic data and study participants’ medical and relevant clinical information. During the interview, study participants were asked to provide written informed consent signed by the patients or their legal guardian. Upon consent given, single 3 to 5 mL blood during an acute phase was drawn from each patient for serum processing. Sera were separated by centrifugation and stored frozen (at −20 °C) until further processing and laboratory analyses were done at the Eijkman Institute for Molecular Biology, Jakarta. The fraction of blood sample from each study participant was analyzed for routine/common blood parameters, i.e., hemoglobin, hematocrit, erythrocyte, platelet, and leucocyte count.

The determination of dengue clinical manifestation was based on WHO-SEARO 2011 guidelines [5]. Patients were classified as having either dengue fever (DF), dengue hemorrhagic fever (DHF), or dengue shock syndrome (DSS). Moreover, patients having unusual manifestations and severe organ involvement, such as of the liver, kidneys, brain, or heart were considered as if they had expanded dengue syndrome. In terms of dengue confirmation, patients with clinical symptoms of dengue and who tested positive for dengue IgM and/or IgG were classified as “probable dengue”, while patients who tested positive for the dengue NS1 antigen and/or RT-PCR were categorized as “confirmed dengue” [5]. Data on the dengue fever IR in Jember, East Java, and Indonesia were compiled from published reports by Jember Regency Health Office, East Java Provincial Health Office and the Ministry of Health of the Republic of Indonesia (*Profil Kesehatan Indonesia*/Indonesia Health Profiles 2014–2019, accessible as data repository at https://pusdatin.kemkes.go.id).

### 2.3. Dengue NS1 Antigen and Serology Tests

Acute serum samples were first screened for the presence of the DENV-encoded Non Structural Protein 1 (NS1) antigen using lateral flow immunochromatographic assays by Standard Q Dengue NS1 Ag Rapid Test (SD Biosensor, Gyeonggi-do, Korea) for dengue confirmation [5]. The presence of anti-dengue IgM and IgG was determined by Standard Q Dengue IgM/IgG Rapid (SD Biosensor). Results from the IgM/IgG tests were used as criteria in defining the infection status (primary or secondary infection). A positive IgM result was indicative of active primary or secondary infection, while an IgG-positive result was indicative of active secondary infection. Primary infection was determined by positive IgM and negative IgG results, while secondary infection was determined by positive IgG, which could be accompanied by a positive IgM result.

### 2.4. Molecular Detection for DENV, Other Arboviruses, and Typhoid Fever

A total amount of 140 µL of serum sample was used for nucleic acid extraction using QIAamp Viral RNA mini kit (Qiagen, Hilden, Germany) according to the manufacturer’s instructions. The extracted RNA was then used as a template in the simultaneous DENV detection and serotyping as well as CHIKV detection performed using abTES DEN/CHIKU 5 qPCR (AIT Biotech, Singapore), according to the method detailed by the manufacturer. The possible presence of flavivirus group pathogens (such as ZIKV and JE viruses) was also screened for using pan-flavivirus real-time qRT-PCR, using primers as described elsewhere [27].

Further screening was done for samples which tested negative for dengue and other arbovirus infection to detect other possible causative pathogens. Analysis for typhoid fever was performed using Tubex TF (IDL Biotech, Bromma, Sweden) based on the Inhibition Magnetic Binding Immunoassay (IMBI) method to detect Salmonella typhi IgM anti-O9 antibodies in serum. The identification of typhoid fever was done following the manufacturer’s recommendations.

### 2.5. DENV Isolation in Cell Culture

Virus isolation approach was performed on serum samples using the cell culture technique. African green monkey (Cercopithecus aethiops) kidney cells (Vero CCL-81 (ATCC)) were seeded at 2 × 10^5^ cells/well of 24-well plate (Corning, Corning, NY, USA) in MEM medium supplemented with 10% of fetal bovine serum (FBS), 2 mM of l-glutamine and 1% antibiotic/antimycotic (all from Gibco, Thermo Scientific, Carlsbad, CA, USA). Plates were incubated overnight at 37 °C inside a humidified incubator with 5% CO_2_ supplementation to generate cell monolayers of 80–100% confluency. An amount of 50 µL of serum sample was prepared in a total of 200 µL of MEM – 2% FBS and inoculated into the designated well containing monolayer of cells. Virus adsorption was allowed for 1 h at 37 °C, 5% CO_2_ before inoculant aspiration and replenishment with fresh medium. Plates were incubated at 37 °C, 5% CO_2_ with daily checking for the presence of cell’s cytopathic effect (CPE), typically for 7–9 days. Supernatant was harvested from wells, centrifuged 3000× *g* for cells and debris separation and stored frozen at −80 °C until use. The presence of DENV was tested for using qRT-PCR molecular detection of extracted RNA.

### 2.6. Determination of DENV Genotype by Envelope Gene Sequencing

The determination of the DENV Genotype was based on the full length envelope (E) gene sequencing using the protocol described earlier [14]. Briefly, cDNA was generated from DENV RNA using Superscript III Reverse Transcriptase (Invitrogen-Thermo Scientific, Carlsbad, CA, USA) and DENV-specific primers. PCR amplification was done using cDNA template and *Pfu* Turbo DNA Polymerase (Stratagene-Agilent Technologies, Santa Clara, CA, USA) to generate PCR products of approximately 2.5 kb in size that were then purified on 0.8% agarose gel using the QIAquick gel extraction kit (Qiagen). Purified amplicons were used as templates in cycle sequencing reactions using BigDye Dideoxy Terminator kits v.3.1 (Applied Biosystems-Thermo Scientific, Foster City, CA, USA) employing overlapping primers for each serotype from both strands [20]. Sequencing was performed on purified DNA using capillary sequencing implemented on a 3130 xl Genetic Analyzer (Applied Biosystems). SeqScape v.2.5 software (Applied Biosystems) was used to assemble the sequence reads, with manual inspection to clarify sequence ambiguities. Sequence alignment was done using a MUltiple Sequence Comparison by Log-Expectation (MUSCLE)rogram inside MEGA X [28] software (v.10.1.1, Penn State University, State College, PA, USA). The classification of the genotypes in each serotype was based on classifications by Goncalvez et al. [29], Twiddy et al. [30], Lanciotti et al. [31], and Lanciotti et al. [32] for DENV-1, -2, -3, and -4, respectively. Initial DENV genotype classification trees are available as Supplementary Figures S1–S4.

### 2.7. DENV Phylogenetic Reconstruction and Evolutionary Analysis

The genetic distance method was used to filter all closely related DENV sequences available in the GenBank repository as of 30 April 2020 implemented in the BLAST website (https://blast.ncbi.nlm.nih.gov). A number of 100 closely related strains from each DENV serotype were used as initial datasets along with Jember isolate sequences. Further filtering was applied to retrieve sequences of the DENV E gene from each serotype with more than 1400 nt. From this, we selected only 60 of the most closely related strains to achieve clarity of the tree view.

The resulting alignment of 1485 nt (1479 nt for DENV-3) was then used in a robust phylogenetic and evolutionary analysis using the Bayesian Markov Chain Monte Carlo (MCMC) algorithm, as implemented in BEAST v.2.6.1 [33]. Datasets were uploaded to the BEAUti v.2.6.1 graphical interface with the isolation year used for the calibration of each taxon. A phylogenetic tree was inferred based on selection of the statistical model for likelihood calculation, using MEGA X [28]. A statistical model was selected based on the Bayesian Information Criterion (BIC) results and the best selected model for all four serotypes datasets was the TrN (TN93) algorithm with four gamma parameters (G4). Molecular clock measurement was set using a relaxed uncorrelated lognormal molecular clock and a Bayesian skyline prior. The analysis was set to generate 100 million chains sampled for every 1000 chains with an initial estimated evolutionary rate set at 7.6 × 10^−4^ substitutions per site per year [34]. The MCMC trace was analyzed using Tracer v.1.7.1 to monitor the adequate Effective Sampling Size (ESS) for all the parameters after 10% burn-in. A maximum clade credibility (MCC) tree was created using TreeAnnotator v.2.6.1 and visualized using FigTree v.1.4.3. The evolutionary parameters were estimated as a median number with 95% Highest Posterior Density (HPD).

### 2.8. Statistical Analysis

Univariate testing was conducted using Pearson’s Chi-square tests for categorical data, while age and hematological data were compared using Kruskal–Wallis tests. Multivariate testing was also conducted using binomial regression analysis for reported symptoms with age, gender, immunologic status, and serotype as potential covariates. All statistical analysis was done using R statistical software (http://www.r-project.org). A probability value of *p* < 0.05 was considered as statistically significant.

## 3. Results

### 3.1. Sample Collection and Demographical Data

**Figure 1:**
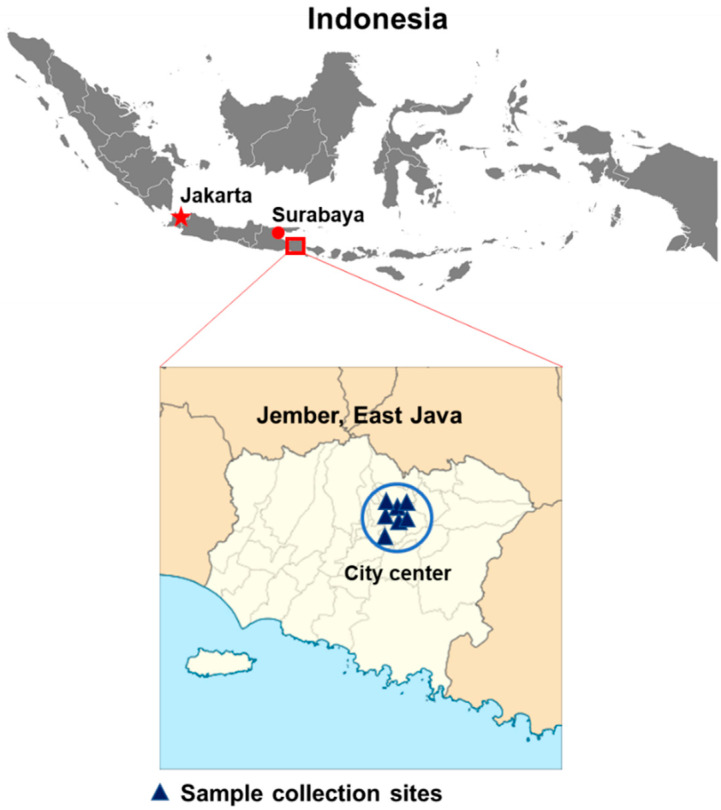
Map of study sites in Jember, East Java province, Indonesia.

**Figure 2:**
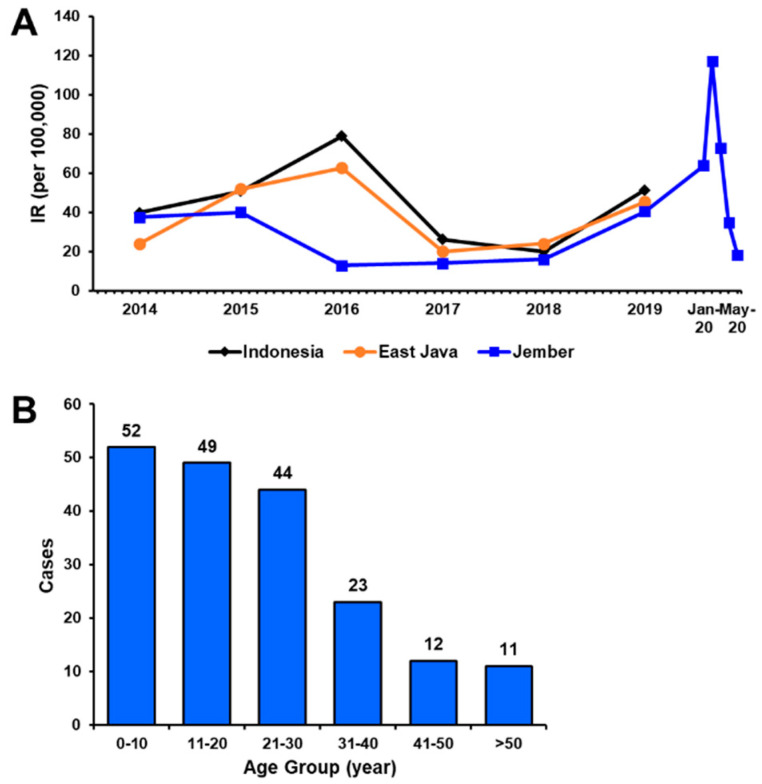
Dengue cases in Jember regency. (**A**) Annual incidence rate (IR) of dengue in Jember regency, East Java province and Indonesia national data, with additional monthly incidence for Jember regency in early 2020 (extrapolated for 12 months). (**B**) Distribution of patient age by groups in Jember in 2019–2020.

From May 2019 to March 2020, a total of 191 febrile patients with dengue-like illness were enrolled at the six collection sites located within the Jember city center (Figure 1). These sites included reference hospitals where people all over Jember regency seek medication for their illness. In general, the annual dengue IR in Jember was lower than that in East Java province and on the national level. The annual IR of dengue in Jember in 2019 was documented at 40.32/100,000 population (Figure 2A). Historically, annual IRs in Jember were relatively stable within the period of three years between 2016 and 2018, a downward-sloping trend from previous years. However, an increasing IR was observed in 2019 and continued with a marked increase from January to March 2020, culminating in a spike in February 2020 with 240 cases (equivalent to an IR of 117.08/100,000 population if extrapolated to 12 months). This sudden increase in dengue incidence was covered in the study period where 55 (or 28.8%) samples were collected during these three months (data not shown).

Based on demographical data collected during patient recruitment, the recruited study participants were aged between 13 months and 64 years with a median age of 20 years (IQR: 9.5–29.0). A total of 98 patients were male (51.3%), with a male-to-female ratio of 1:0.95. Among all of the suspected dengue patients, children aged 0–10 years account for the highest proportion (27.2%), followed by adolescents 11–20 years old (25.7%) and young adults 21–30 y.o (23.0%) (Figure 2B). More children than adults were suspected of having dengue in Jember.

### 3.2. Clinical Examination of Dengue-Suspected Patients

We used clinical examination data to study the most common symptoms suffered by all dengue-suspected patients. Among all 191 patients, headache was the most common symptom reported (83.2%), followed by myalgia (52.4%) and vomiting (41.9%) (Table 1). Bleeding manifestation was observed in 14.1% of patients. However, none were reported to have other signs of severe dengue such as pleural effusion, ascites, and altered consciousness. Moreover, there was no significant correlation observed between reported symptoms and potentially affecting covariates, such as age, gender, infection status, and the infecting DENV serotype (Supplementary Table S1).

### 3.3. Dengue Serological Testing and Case Confirmation

**Figure 3:**
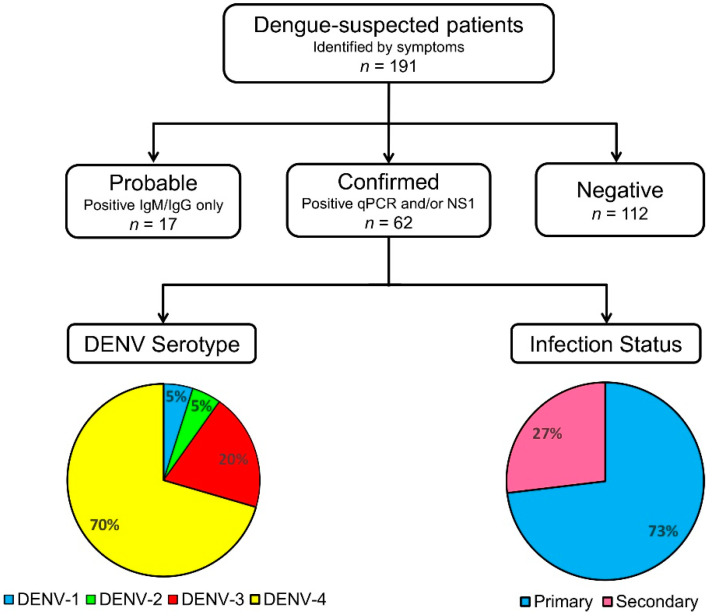
The samples processing flow implemented in Jember dengue study.

Among all 191 dengue-suspected patients identified by symptoms, we found that 17 samples (8.9%) were probable dengue cases while another 62 samples (32.5%) were confirmed as dengue by NS1 antigen detection and/or molecular testing (Figure 3). Combined together, the burden of dengue in Jember was 41.4% (79/191). Out of the 62 confirmed dengue patients, 25 (40.3%) tested positive for both NS1 and via RT-PCR. The other 36 (58.1%) samples tested positive via RT-PCR only, and 1 sample (1.6%) tested positive for NS1 only.

When looking at the clinical manifestations of 79 probable and confirmed dengue patients, the majority (58 or 73.4%) manifested as the milder DF clinical manifestation, while the other 21 (26.6%) patients had the more severe DHF manifestation. Based on the infection status, a higher percentage of primary infection (73.4%) was recorded than secondary infections (26.6%). There was no correlation between patients’ age and gender and infection status. However, secondary infections were more likely to present with DHF compared to primary infection (*p* = 0.005) (Table 2). Examining the laboratory data, the median platelet count of patients with secondary infections is significantly lower (*p* < 0.001) than that of primary infections. Out of 58 primary dengue infections, 10 were categorized as DHF due to severe thrombocytopenia of 50 × 10^3^/µL counts or less, rapidly decreasing platelet counts, or an increase in hematocrit levels of 20% or more.

### 3.4. DENV Circulating in Jember

Among 62 dengue-confirmed samples, the serotype of DENV could be determined from 61 samples using RT-PCR, leaving one sample with a negative RT-PCR result but positive for the NS1 antigen. All four serotypes of DENV were detected with the predominance of DENV-4 in 43 (70.5%) of the samples, followed by 12 DENV-3 (19.7%), and three samples (4.9%) for both DENV-1 and DENV-2 (Figure 3). Within the time period between January and March 2020, where a sudden increase in cases was observed, a total number of 12 samples tested positive by RT-PCR and serotyping determined the presence of DENV-1, -2, -3, and -4 in two (16.7%), three (25.0%), three (25.0%), and four (33.3%) samples, respectively.

### 3.5. DENV Serotypes and Their Clinical Correlates

There was no correlation between the infecting DENV serotypes and patient’s age, gender, clinical manifestation, and infection status. Platelet count was found to be correlated with DENV serotypes (*p* = 0.008) with post hoc analysis showing the lower platelet count in DENV-1 samples (*p* = 0.019) (Table 3). The median platelet count was also significantly lower in patients with secondary infections than in those with primary infections (*p* < 0.001, data not shown). This was in line with the result among probable and confirmed patients (Table 2).

### 3.6. Non-DENV Arboviruses and Other Pathogens Circulating Jember

Testing for viruses other than DENV was conducted for the possible CHIKV and other flavivirus infections. From all 191 samples tested, we did not find any CHIKV infection. Further testing using pan-flavivirus RT-PCR yielded 11 (5.8%) positively detected samples (data not shown). To determine the identity of those flavivirus PCR amplicons, Sanger capillary sequencing was performed using pan-flavivirus primers. All pan-flavivirus-positive samples were confirmed as DENVs.

Tubex testing for typhoid fever diagnosis was also done on 169 samples, 32 (18.9%) of which were positive. Out of those 32 samples, 11 samples also tested positive for DENV and 4 samples also tested positive for dengue antibodies. The remaining 95 samples that were negative for DENV, CHIKV, and had negative or unavailable Tubex results were categorized under “fever of unknown origin (FUO)”. Patients with dengue, typhoid fever, and fever of unknown origin report very similar symptoms. However, when comparing hematology results, cases of dengue infections present with lower platelet counts (Table 4).

### 3.7. DENV Genotypes in Jember

We have successfully generated 22 full-length E gene sequences from Jember DENV isolates. The sequences were deposited in the GenBank repository, with accession numbers shown in Table 5. The full-length E gene sequence is 1485 nt for DENV-1, -2, and -4, and 1479 nt for DENV-3.

**Figure 4:**
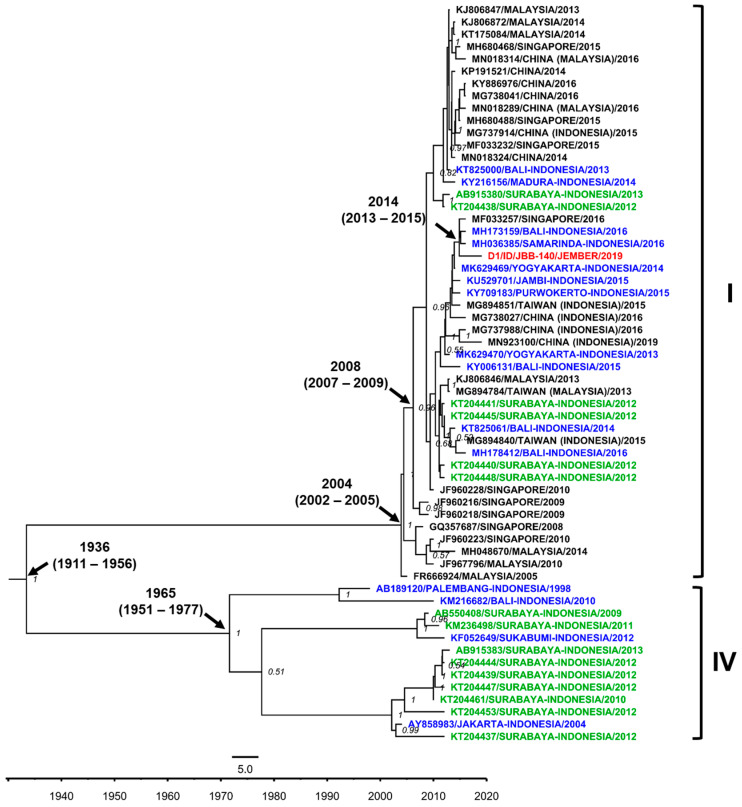
The Maximum Clade Credibility (MCC) phylogenetic tree of DENV-1 genotype I and IV strains generated by BEAST Bayesian inference method with TrN + G evolution model calculated using E gene sequences. The red labels indicate isolates from Jember, while blue labels indicate strains from other cities in Indonesia and green labels indicate strains from the nearby Surabaya city. The number in the node indicates the posterior probability of that particular cluster, with values higher than 0.5 shown. Time to the most recent common ancestor (tmrca) is indicated as median year (95% Highest Posterior Density (HPD)).

We were only able to sequence one DENV-1 isolate from Jember. The isolate was grouped into the Genotype I of DENV-1 and showed close-relatedness to strains from Samarinda, East Kalimantan [35], and other strains from cities in Indonesia, i.e., Bali [36], Yogyakarta [37], Jambi [38], and Purwokerto, Central Java [39], along with strains from imported cases to Taiwan and China and an isolate from Singapore (Figure 4). The Jember DENV-1 isolate was likely becoming the basal taxon for clade-consisting strains from Samarinda, Bali, and Singapore, with the time to the most recent common ancestor (tmrca) estimated in 2014. Within the larger clade, Surabaya strains were also grouped. The Genotype I was estimated to emerge circa 2004. Unlike the previous studies by our group and others in Surabaya, the presence of Genotype IV in Jember was not detected.

**Figure 5:**
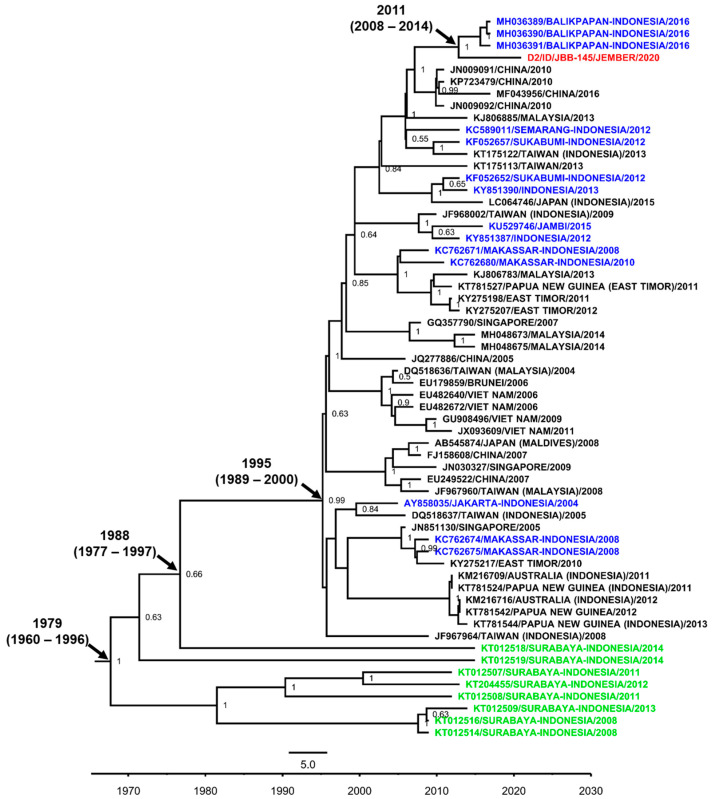
The Maximum Clade Credibility (MCC) phylogenetic tree of DENV-2 Cosmopolitan genotype generated by BEAST Bayesian inference method with TrN + G evolution model calculated using E gene sequences. The red labels indicate isolates from Jember while blue labels indicate strains from other cities in Indonesia and green labels indicate strains from nearby Surabaya city. The number in the node indicates the posterior probability of that particular cluster, with values higher than 0.5 shown. Time to the most recent common ancestor (tmrca) is indicated as median year (95% Highest Posterior Density (HPD)).

Similarly, only one isolate of DENV-2 was successfully sequenced and genetically grouped to the Cosmopolitan genotype of DENV-2. This isolate from Jember was forming a monophyletic group with Indonesian strains from Balikpapan, East Kalimantan [35] with the tmrca estimated at 2011 (Figure 5). However, we observed that the isolate was relatively distinct to DENV-2 from other cities in Indonesia. Moreover, the Jember isolate was quite distinct and evolutionarily separated for years from Surabaya strains.

**Figure 6:**
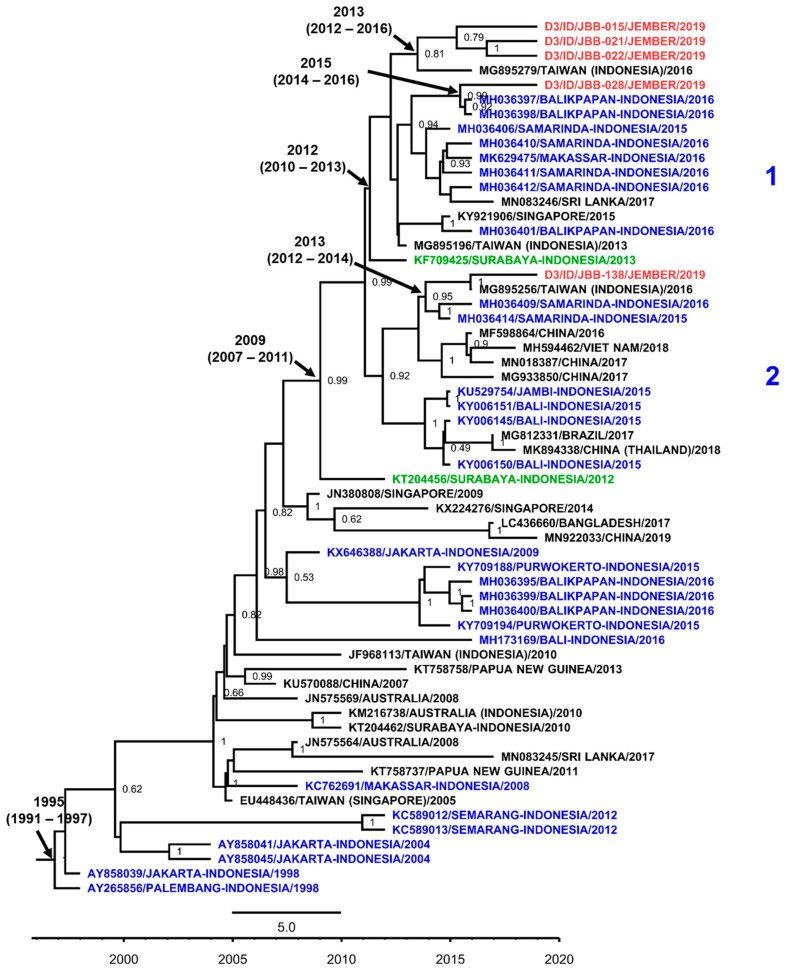
The Maximum Clade Credibility (MCC) phylogenetic tree of DENV-3 Genotype I generated by BEAST Bayesian inference method with a TrN + G evolution model calculated using E gene sequences. The red labels indicate isolates from Jember, while blue labels indicate strains from other cities in Indonesia and green labels indicate strains from nearby Surabaya city. The number in the node indicates the posterior probability of that particular cluster, with values higher than 0.5 shown. Arabic numbers depict further grouping into DENV lineages. Time to the most recent common ancestor (tmrca) is indicated as median year (95% Highest Posterior Density (HPD)).

Among 12 samples detected as DENV-3, we successfully sequenced the E gene of five (41.7%) isolates. The DENV-3 isolates from Jember were classified as Genotype I (Figure 6). Further grouping into clades were detected with the first clade consisting of four Jember isolates grouped together in a monophyletic clade supported by a strong posterior, which emerged circa 2013 to 2015. These isolates were closely related to strains from the Indonesian cities of Samarinda and Balikpapan, East Kalimantan [35] and Makassar, South Sulawesi [17], and strains related to dengue cases in Taiwan. The second clade consists of one isolate of Jember DENV-3, which also shared a relationship with strains from East Kalimantan and a strain from an imported case in Taiwan with a tmrca of around the year 2013. A strain from Surabaya is located in the basal position of the first clade and shared the same common ancestor as a strain which emerged around 2012. Another Surabaya strain was also identified in the basal position, with a tmrca estimated to be in 2009, sharing the common ancestor with both clades where Jember isolates were located.

**Figure 7:**
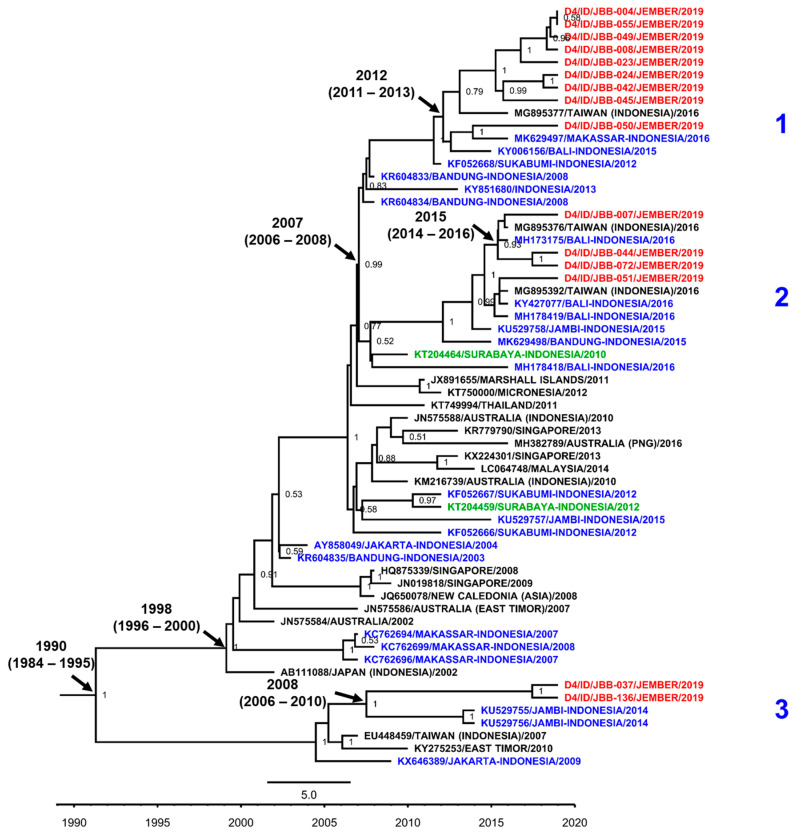
The Maximum Clade Credibility (MCC) phylogenetic tree of DENV-4 Genotype II generated by BEAST Bayesian inference method with TrN + G evolution model calculated using E gene sequences. The red labels indicate isolates from Jember, while blue labels indicate strains from other cities in Indonesia and green labels indicate strains from nearby Surabaya city. The number in the node indicates the posterior probability of that particular cluster, with values higher than 0.5 shown. Arabic numbers depict further grouping into DENV lineages. Time to the most recent common ancestor (tmrca) is indicated as median year (95% Highest Posterior Density (HPD)).

As the predominant serotype, all DENV-4 isolates from Jember were grouped into Genotype II (Figure 7). We have generated sequences from 15 out of 43 (34.9%) isolates confirmed as DENV-4. Further grouping into three monophyletic clades were observed. Clade 1 consists of nine Jember DENV-4 isolates grouped together with a strain of imported cases to Taiwan and other strains from cities in Indonesia, i.e., Makassar, Bali [40], and Sukabumi, West Java [41]. These taxa were estimated to have a tmrca around 2012. The second clade grouped four Jember DENV-4 isolates along with strains from Taiwan, and the Indonesian cities of Bali and Jambi. This clade was estimated to have a younger tmrca in 2015. The other clade—Clade 3—showed a relatively distinct grouping with two Jember isolates displaying a close relationship with strains from Jambi. The estimated tmrca for this particular clade is around 2008.

The molecular clock analysis inferred the evolutionary parameters for DENV isolates from Jember. The age for each DENV serotype tree was estimated at 83, 41, 24, and 29 years for DENV-1, DENV-2, DENV-3, and DENV-4, respectively. Higher evolutionary rates were recorded for DENV-3 and DENV-4 trees (Table 6). However, the rates were still within the normal range of DENV evolutionary rates [34]. Looking at Jember isolates, the evolutionary rate of the DENV isolates were similar, or within the range of, the median rate of the trees. However, DENV-3 isolates from Jember showed distinctively high evolutionary rates above the range of the DENV-3 tree.

## 4. Discussion

Dengue and other arbovirus molecular surveillance studies were performed in Jember regency, the third largest city in East Java Province, Indonesia. This 11-month study aimed to monitor the transmission of DENV and other arboviruses in the region, also describing the characteristics of dengue outbreak based on clinical, serological, and viral genetic data. Such monitoring and detailed analysis of clinical cases is essential in less-developed, and thus under-studied, areas such as Jember, as there is lack of historical data documenting dengue in this urban city.

Within the 11-month period, a total of 191 samples of dengue-suspected patients were collected. Although lower dengue IR in Jember was documented compared to East Java province and national rates, there was a sudden increase of dengue incidence in the early months of 2020 (Figure 2A). Looking deeper into this time period, the dengue cases peaked in February 2020. Nearly 30% of our samples were collected in the 3-month period around this peak (from January to March). Moreover, from those 55 samples, there are 12 (21.8%) samples which tested positive using RT-PCR with all four DENV serotypes detected. The persistent incidence of dengue in Jember, the routine annual outbreak happening in the regency, and the sudden increase of cases during the monsoon rainy season, need further investigation.

The majority of patients attending six sample collection sites were children under 10 y.o (Figure 2B). This is similar to our previous dengue disease data gathered in Surabaya [14]. The patients were seeking clinical attention mostly due to symptoms of headache, myalgia, and vomiting, with some patients having bleeding manifestations. Looking at the dengue burden in Jember, we observed a percentage of 41.4% burden with confirmed cases making up 32.5%, reflecting the relatively high burden imposed on the Jember population. Out of DENV-negative samples, we did not detect any other arbovirus infections. This may be due to the low prevalence of Chikungunya [22,42] and Zika [25] infections in Indonesia. However, we detected cases of typhoid fever with a positivity of 18.9%, including those also infected with DENV, with very similar reported symptoms and laboratory results. This finding confirms that typhoid fever is considered to be one of the causative agents of febrile illness and should be considered in the clinical setting as a differential or an additional diagnosis of dengue [43].

Exploring the clinical data documented during the study, we observed that more patients presented with milder DF compared to DHF manifestations. Furthermore, when looking at the infection status, the higher percentage of primary infection was evident. The pattern of milder dengue manifestations being correlated with primary infection was also reported in a dengue surveillance study in Jambi in 2015 [38]. On the other hand, secondary infections were more likely to present with DHF manifestation in Jember (Table 2). This is consistent with reports describing the correlation between secondary infection and the more severe manifestation of dengue in patients [44]. Moreover, secondary infection is correlated with a lower platelet count in Jember patients. It has been reported that platelet-associated IgG (PAIgG) formation involving anti-dengue virus IgG induces transient thrombocytopenia during the acute phase of secondary dengue virus infection [45], which may explain the correlation observed.

All four serotypes of DENV were found to be circulating in Jember. Strikingly, we found a predominance of DENV-4 with some additional DENV-3 cases, along with a few cases of DENV-1 and DENV-2 (Figure 3). While DENV-4 is known to circulate in East and South East Asia [9], analysis of previous dengue outbreaks in Indonesia have almost exclusively highlighted DENV-1 to -3 as the causative pathogens [12,14,17,19,20], with only one report of an outbreak in Jakarta based on a limited number of cases [21]. Furthermore, the predominant serotype in the nearby city of Surabaya in 2012 was documented as DENV-1 [14,15]. Here, we observed a different serotype distribution in Jember. This is the first data of serotype distribution in Jember, since there is no historical published data for this regency.

The epidemic of DENV-4 in Jember may be caused by several possible considerations. First, this serotype may have been newly introduced to the area from other regions in Indonesia [46]. The low herd immunity in Jember (26.6%) may easily have facilitated the local transmission of this “benign” serotype. Secondly, this serotype may have re-emerged from local endemic viruses, as previously described in Jakarta in the case of DENV-3 [46]. Another alternative is the occurrence of a serotype switch, as has happened in the nearby city of Surabaya [12,13]. The introduction of a new DENV serotype in a population with a low herd immunity would lead to a high ratio of primary to secondary infections, even in an area in which other serotypes commonly circulate. This seems to be the case here, as there is an overrepresentation of primary infections in this outbreak (73.1%), demonstrating a relatively low dengue herd immunity in the area. By comparison, other studies of DENV outbreaks in Java, such as those in Semarang and Jakarta, found the rates of primary infections to be only 23% and 18.7% respectively [20,47], showing the hyperendemicity of DENV across the island. All four DENV serotypes were detected in our study, which may lead to future outbreaks due to serotype-shift. Since secondary infections are known to lead to more severe clinical manifestations than primary infections due to antibody-dependent enhancement (ADE), possible future outbreaks in Jember may lead to larger proportions of the population with severe disease. Interestingly, despite the widespread nature of DENV-3 in Java, all of the confirmed cases of this serotype in this study were primary infections (Table 3). This indicates that there may not have been a consistent circulation of this serotype in the regency for the past few years, long enough for existing immunity to be lost [48,49]. Following this absence of DENV-3, a recent reintroduction of the virus would provide an explanation for the new primary infections.

Molecular surveillance of DENV is essential in a case such as this, as the emergence of new serotypes, genotypes, or lineages of DENV or the introduction of one of these into a naïve population has been shown to significantly increase the incidence of dengue, as well as the severity of the symptoms [16,29]. There is also some data to suggest that different serotypes are linked to a varying severity of the disease [50,51,52]. While our data did not show a significant correlation between serotype and disease severity (Table 3), this may be attributable to the low number of cases of DENV-1 and -2. However, the fact that DENV-4 was the predominant serotype during the epidemic of mild dengue in Jember is in line with the opinion that considered DENV-4 as the most mild of the four DENV serotypes [4].

Phylogenetic analyses revealed the close relationship between Jember isolates and strains from other cities in Indonesia. Surprisingly, the genetic distance analysis revealed that there was no direct relationship between Jember isolates and strains from Surabaya, the nearest large urban city to Jember in the East Java province. The DENV isolates from Jember were likely to be more related to strains from Samarinda and Balikpapan, both cities in East Kalimantan, from a dengue surveillance study conducted in recent years [35]. Further connections between these cities may need to be explored more extensively. However, traces of transmission and the influence of Surabaya city on Jember may be seen in the presence of Surabaya strains in the basal position of clades leading to Jember isolates. Together, the data shows that the Jember DENV isolates were local endemic viruses circulating in the region, with DENV-4 re-emerging, likely due to a low herd immunity.

The limitations of this study were related to the sampling sites, which were focused in the Jember city center area and were not spread throughout all the areas of the regency. The distinct proportion of DENV serotypes also lowered the power of the statistical analysis, especially for DENV-1 and DENV-2 samples. Moreover, we did not assess the contribution of mosquito vectors on the transmission of dengue in Jember. Nevertheless, our data provides important epidemiological information that will be useful for future surveillance.

In summary, we reveal the first comprehensive clinical and virological data of the DENV-4 outbreak in Indonesia in the Jember regency. Our study highlights the correlation of this outbreak with primary infections and milder forms of dengue. Continuous dengue surveillance is important to have more complete data on dengue in Jember and surrounding areas and to increase preparedness for potential outbreaks in the future.

## Figures and Tables

**Table 1:** Reported symptoms of acutely febrile dengue-suspected patients.

Symptoms (*n* = 191)	*n* (%)
Headache	159 (83.2)
Myalgia	100 (52.4)
Vomiting	80 (41.9)
Stomachache	73 (38.2)
Arthralgia	69 (36.1)
Retro-orbital Pain	47 (24.6)
Bleeding manifestation	27 (14.1)
Rash	13 (6.8)
Positive Tourniquet Test	10 (5.2)
Altered Consciousness	0 (0)
Ascites	0 (0)
Pleural Effusion	0 (0)

**Table 2:** Characteristics of dengue among probable and confirmed patients in term of infection status.

Parameters	Infection Status (*n* = 79)		*p*-Value ^a^
	Primary (*n* = 58)	Secondary (*n* = 21)	
*Age*, Median (IQR)	19.5 (8–28.5)	19 (9–26)	0.665 ^b^
*Age groups*			1.000
Children (<20 y.o)	28	10	
Adults (≥20 y.o)	30	11	
*Gender*			0.111
Male	33	7	
Female	25	14	
*Clinical manifestation*			**0.005**
DF	48	10	
DHF	10	11	
*Hematology data*, Median (IQR)			
Platelet (×10^3^/µL)	172 (105.3–223)	57 (35–85)	**<0.001 ^b^**
Hematocrit (%)	41.1 (38.3–44.8)	42.9 (41.3–46.0)	0.052 ^b^
WBC (×10^3^/µL)	4.3 (3.2–7.3)	3.6 (2.7–5.6)	0.502 ^b^

IQR, interquartile range; WBC, white blood count ^a^ Pearson’s Chi-squared test ^b^ Kruskall–Wallis test. Statistically significant values are shown in bold.

**Table 3:** Jember DENV serotypes and their clinical correlates.

Parameters	DENV Serotype (*n* = 61)				*p*-Value
	DENV-1 (*n* = 3)	DENV-2 (*n* = 3)	DENV-3 (*n* = 12)	DENV-4 (*n* = 43)	
*Age*, Median (IQR)	8	11	20	20	0.336 ^a^
	(7–12.5)	(7.2–20)	(11.8–22.3)	(11.5–30.5)	
*Gender*					0.143 ^b^
Male	3	1	4	25	
Female	0	2	8	18	
*Clinical manifestation*					0.011 ^b,c^
DF	0	1	10	33	
DHF	3	2	2	10	
*Infection status*					0.004 ^b,d^
Primary	1	1	12	36	
Secondary	2	2	0	6	
*Hematology*, Median (IQR)					
Platelet (×10^3^/µL)	20	73	185	143	**0.008 ^a,e^**
	(15.5–20.5)	(53.5–86)	(118.8–194)	(76–227.5)	
Hematocrit (%)	2.90	2.60	3.65	4.64	0.529 ^a^
	(2.65–3.25)	(2.50–3.20)	(2.90–4.60)	(2.95–8.50)	
WBC (×10^3^/µL)	47.5	44.4	42.0	41.5	0.204 ^a^
	(43.2–47.8)	(41.6–45.2)	(38.3–45.6)	(38.6–45.1)	

IQR, interquartile range. ^a^ Kruskall–Wallis test. ^b^ Pearson’s Chi-squared test. ^c^ Post-hoc Bonferroni: *p* = 0.112 (DENV-1—others), *p* = 1.000 (DENV-2—others), *p* = 1.000 (DENV-3 – others), *p* = 1.000 (DENV-4—others). ^d^ Post-hoc Bonferroni: *p* = 0.338 (DENV-1—others), *p* = 0.338 (DENV-2—others), *p* = 0.776 (DENV-3—others), *p* = 1.000 (DENV-4—others). ^e^ Post-hoc Dunn: *p* = 0.019 (DENV-1—others), *p* = 0.438 (DENV-2—others), *p* = 0.660 (DENV-3—others), *p* = 1.000 (DENV-4—others).

**Table 4:** Clinical features of dengue, typhoid fever, double infections, and fever of unknown origin (FUO).

*n* (%)	Dengue (*n* = 64)	Typhoid (*n* = 17)	Double Dengue-Typhoid (*n* = 15)	FUO (*n* = 95)	Pearson’s Chi Square Test
Headache	50 (78.1)	14 (82.4)	14 (93.3)	81 (85.3)	0.404
Retro-orbital Pain	17 (26.6)	4 (23.5)	1 (6.7)	25 (26.3)	0.402
Myalgia	35 (54.7)	9 (52.9)	4 (26.7)	52 (54.7)	0.218
Arthralgia	25 (39.1)	5 (29.4)	2 (13.3)	37 (38.9)	0.225
Rash	3 (4.7)	0 (0)	0 (0)	10 (10.5)	0.182
Stomachache	27 (42.2)	7 (41.2)	4 (26.7)	35 (36.8)	0.713
Vomiting	23 (35.9)	10 (58.8)	6 (40.0)	41 (43.2)	0.382
Bleeding	8 (12.5)	4 (23.5)	3 (20.0)	12 (12.6)	0.577
Positive Tourniquet	11 (17.2)	0 (0)	2 (13.3)	7 (7.4)	0.097
					One-Way ANOVA
Platelet (×10^3^/µL)	140 (71–223)	174 (135–223)	136 (74–181)	171 (138–254)	0.005 *
Hematocrit (%)	42 (38.9–45.0)	36 (34.2–41.0)	42 (35.5–45.6)	40 (36.6–44.0)	0.249
WBC (×10^3^/µL)	4.4 (2.85–7.13)	5.6 (4.63–7.10)	4.0 (3.25–6.05)	5.6 (4.22–8.35)	0.119

* Post-hoc Dunn Test: *p* = 0.701 (Dengue vs. Double Infection), *p* = 0.016 (Dengue vs. FUO), *p* = 0.062 (Double Infection vs. FUO), *p* = 0.299 (Dengue vs. Typhoid), *p* = 0.304 (Double Infection vs. Typhoid), *p* = 0.779 (FUO vs. Typhoid).

**Table 5:** Samples with sequenced DENV E genes and their genotype grouping.

No.	Sample ID	Serotype	Genotype	Age (y)	Manifestation	Accession No.
1.	JBB-140	DENV-1	I	8	DHF	MT377728
2.	JBB-145	DENV-2	Cosmopolitan	4	DHF	MT377729
3.	JBB-015	DENV-3	I	1	DF	MT377730
4.	JBB-021	DENV-3	I	8	DF	MT377731
5.	JBB-022	DENV-3	I	2	DF	MT377732
6.	JBB-028	DENV-3	I	21	DF	MT377733
7.	JBB-138	DENV-3	I	35	DHF	MT377734
8.	JBB-004	DENV-4	II	40	DF	MT377735
9.	JBB-007	DENV-4	II	16	DHF	MT377736
10.	JBB-008	DENV-4	II	26	DF	MT377737
11.	JBB-023	DENV-4	II	38	DF	MT377738
12.	JBB-024	DENV-4	II	7	DF	MT377739
13.	JBB-037	DENV-4	II	62	DF	MT377740
14.	JBB-042	DENV-4	II	19	DHF	MT377741
15.	JBB-044	DENV-4	II	18	DF	MT377742
16.	JBB-045	DENV-4	II	12	DF	MT377743
17.	JBB-049	DENV-4	II	18	DF	MT377744
18.	JBB-050	DENV-4	II	11	DHF	MT377745
19.	JBB-051	DENV-4	II	8	DHF	MT377746
20.	JBB-055	DENV-4	II	42	DF	MT377747
21.	JBB-072	DENV-4	II	5	DF	MT377748
22.	JBB-136	DENV-4	II	38	DF	MT377749

**Table 6:** Evolutionary rates of DENV from Jember.

Serotype	DENV Tree			Jember DENV Isolate(s)	
	*n*	Tree Age (Years)	Median Rate × 10^−4^ (95% HPD)	*n*	Mean Rate (×10^−4^) ± STDEV
DENV-1	60	83	7.7 (5.9–9.7)	1	7.7
DENV-2	60	41	6.5 (4.2–9.4)	1	7.2
DENV-3	60	24	11.2 (9.0–13.4)	5	15.5 ± 4.2
DENV-4	60	29	10.0 (8.2–11.8)	15	9.9 ± 0.17

## Supplementary Materials

The following are available online at https://www.mdpi.com/1999-4915/12/9/913/s1, Table S1: Multivariate analysis of dengue symptoms and the potentially affecting covariates, Figure S1: Phylogenetic tree and genotype classification of DENV-1 isolate from Jember, East Java (marked with red dot) with other reference strains, Figure S2: Phylogenetic tree and genotype classification of DENV-2 isolate from Jember, East Java (marked with red dot) with other reference strains, Figure S3: Phylogenetic tree and genotype classification of DENV-3 isolates from Jember, East Java (marked with red dots) with other reference strains, Figure S4: Phylogenetic tree and genotype classification of DENV-4 isolates from Jember, East Java (marked with red dot) with other reference strains.
