# Supplementary material for: Dengue Virus Serotype 4 Is Responsible for the Outbreak of Dengue in East Java City of Jember, Indonesia
Source: Viruses. 2020 Aug 20;12(9):913. doi: 10.3390/v12090913 (PMC7551817; doi:10.3390/v12090913)
Supplement: Supplementary file 1 [file viruses-12-00913-s001.zip › viruses-862245 supplementary/viruses-862245 supplementary table.docx]

**Supplementary Table 1.** Multivariate analysis of dengue symptoms and the potentially affecting covariates.

| **Parameters** | **Binomial logistic regression, p-value** | | | | | |
| --- | --- | --- | --- | --- | --- | --- |
|  | **Age** | **Gender (Female)** | **Infection Status (Secondary)** | **Serotype (DENV-2)** | **Serotype (DENV-3)** | **Serotype (DENV-4)** |
| Headache | 0.664 | 0.349 | 0.513 | 0.994 | 0.592 | 0.578 |
| Myalgia | 0.148 | 0.715 | 0.281 | 0.335 | 0.761 | 0.453 |
| Vomiting | 0.239 | 0.783 | 0.329 | 0.991 | 0.990 | 0.990 |
| Stomachache | 0.319 | 0.909 | 0.069 | 0.993 | 0.757 | 0.681 |
| Arthralgia | 0.181 | 0.838 | 0.279 | 0.355 | 0.796 | 0.241 |
| Retro-orbital Pain | 0.397 | 0.889 | 0.300 | 1.000 | 0.993 | 0.994 |
