# Supplementary figures and images for: Dengue Virus Serotype 4 Is Responsible for the Outbreak of Dengue in East Java City of Jember, Indonesia
Source: Viruses. 2020 Aug 20;12(9):913. doi: 10.3390/v12090913 (PMC7551817; doi:10.3390/v12090913)

# Supplementary Figure 1

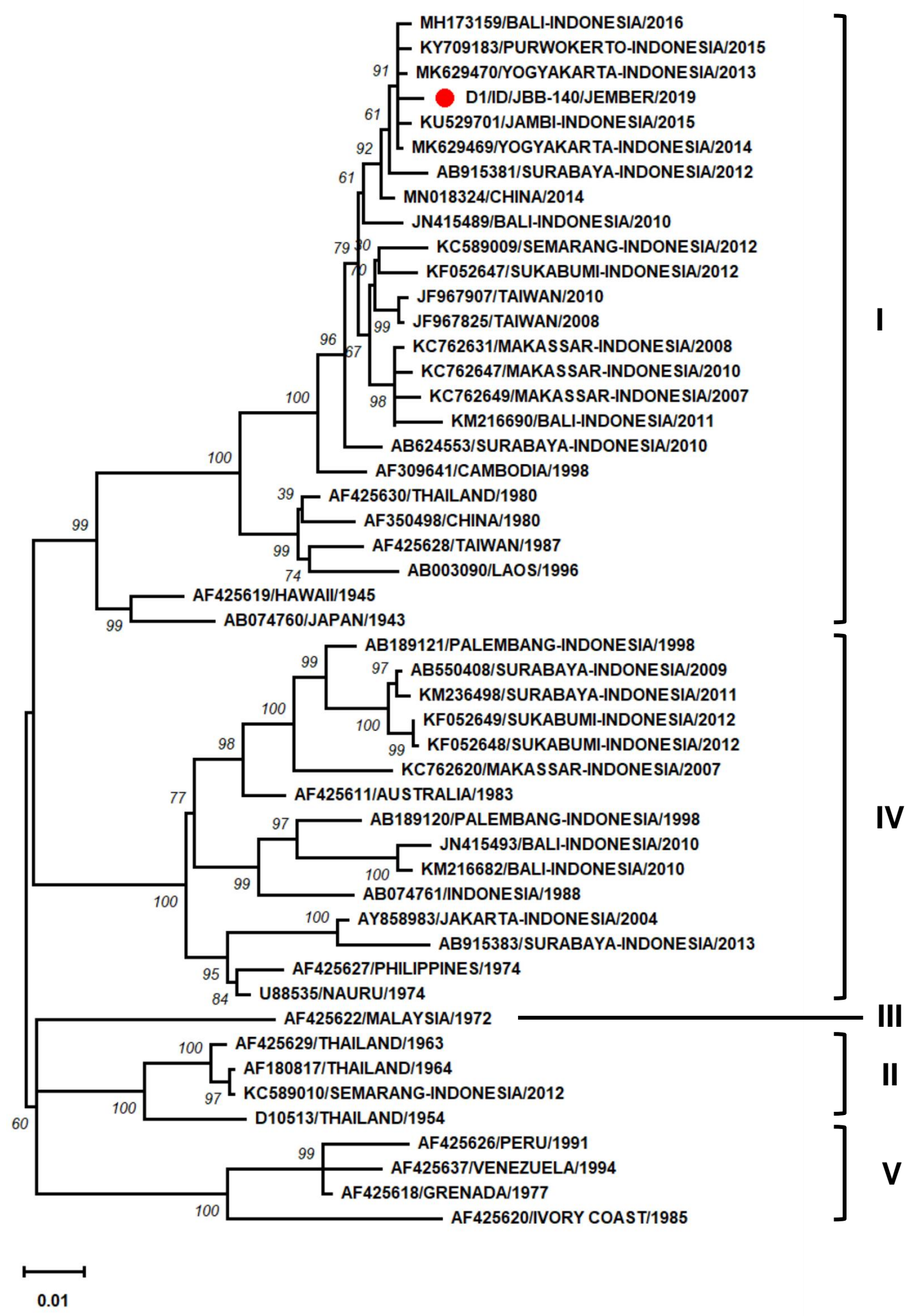

# Supplementary Figure 2

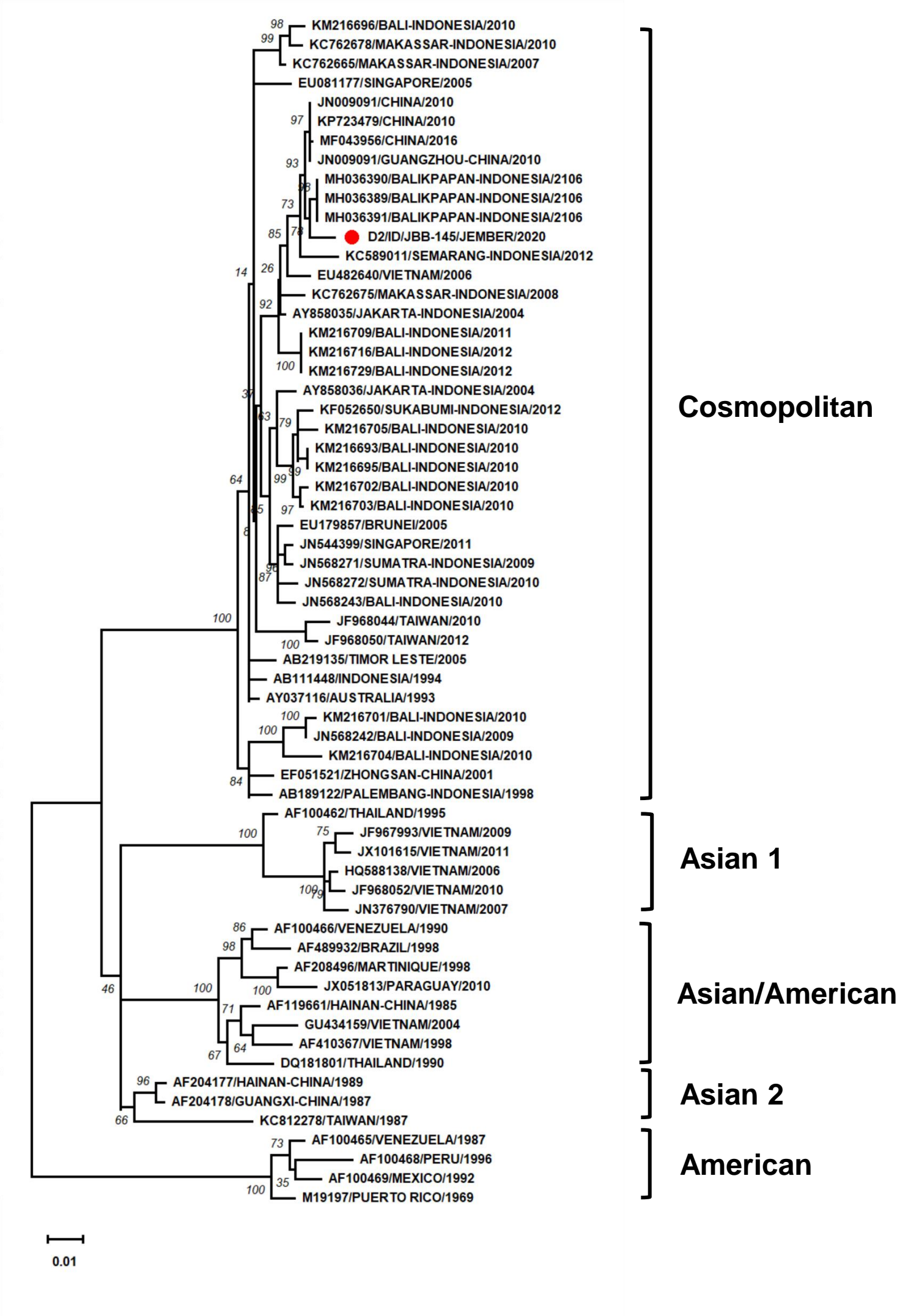

# Supplementary Figure 3

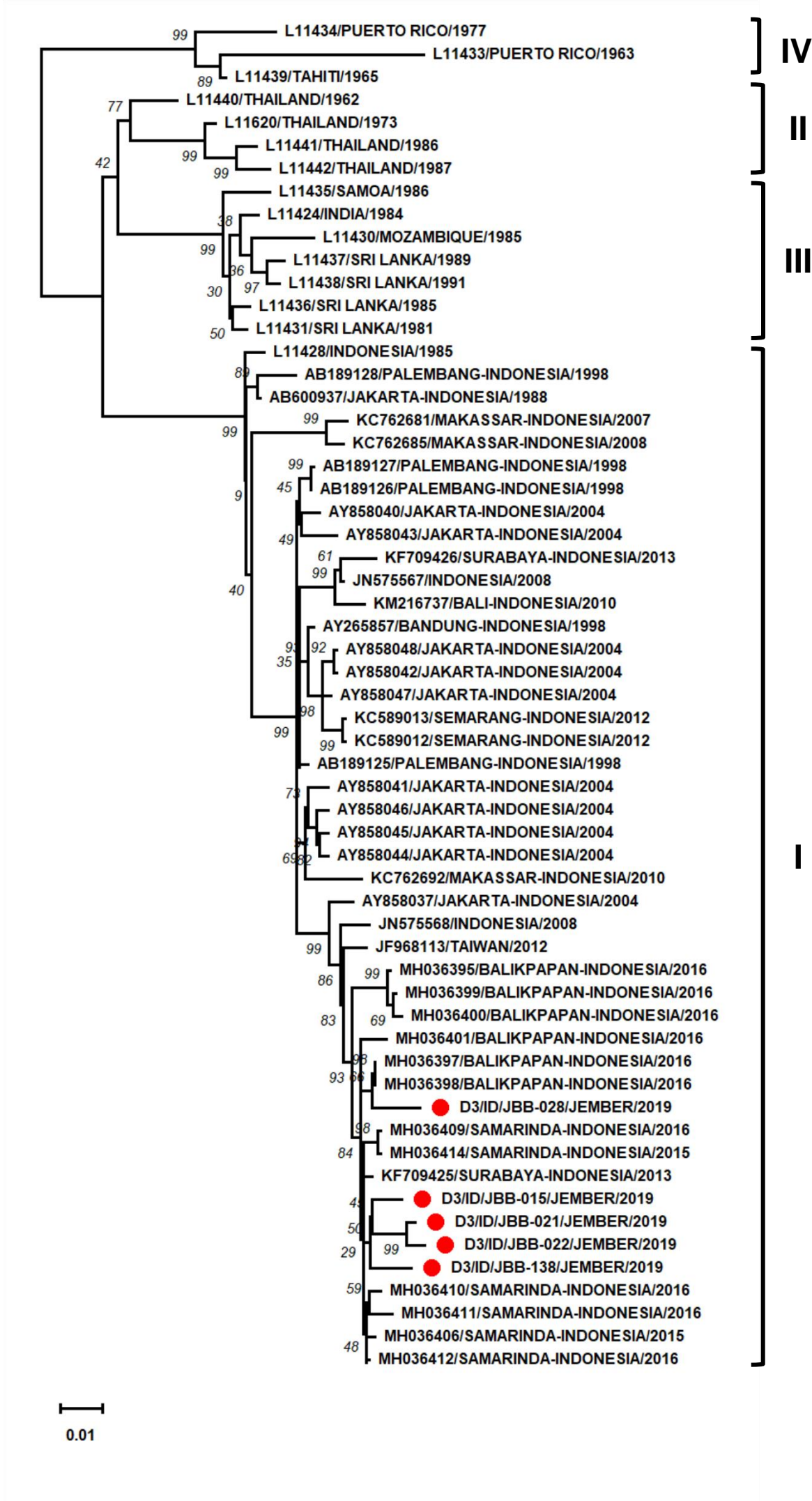

# Supplementary Figure 4

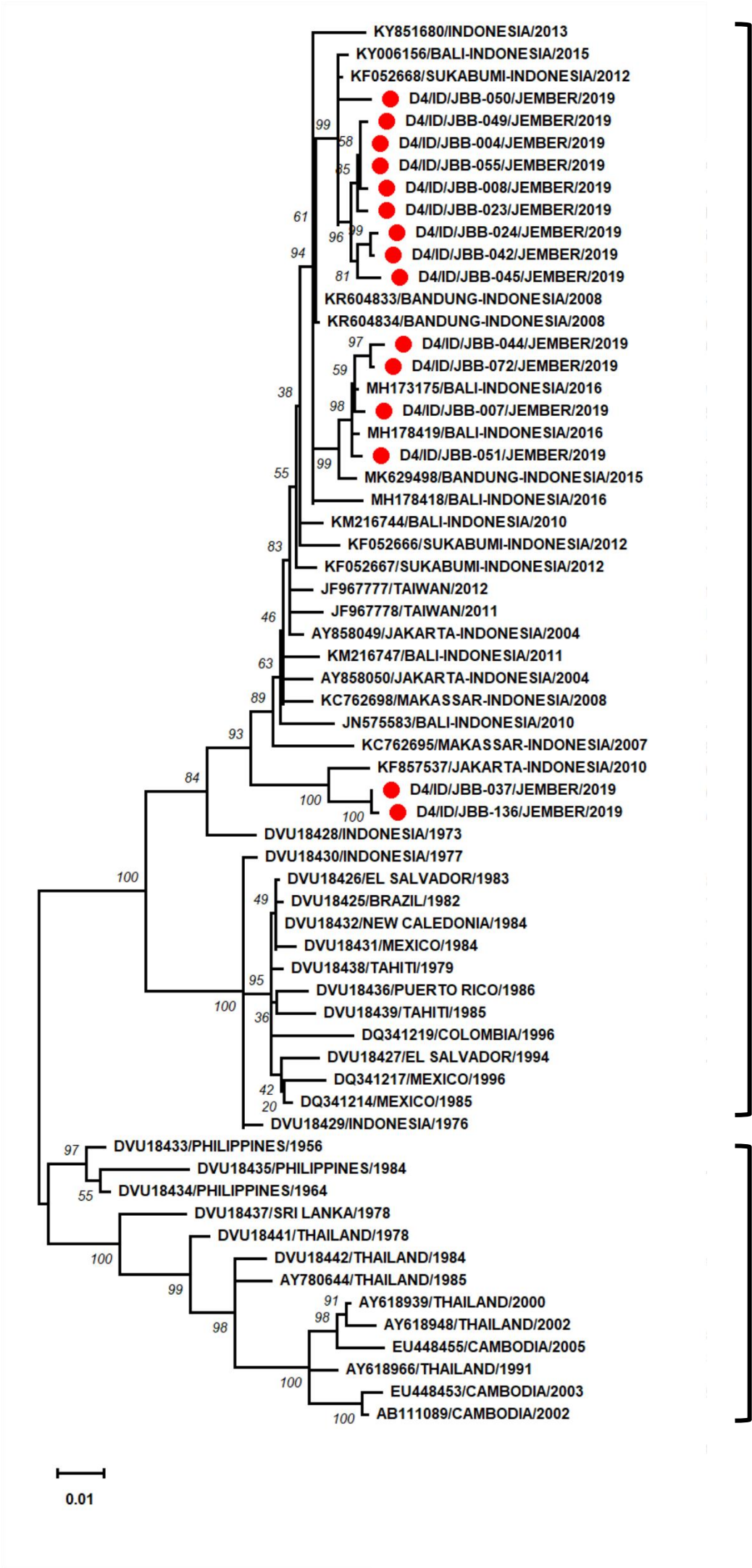

Supplement: Supplementary file 1 [file viruses-12-00913-s001.zip › viruses-862245 supplementary/viruses-862245 supplementary figures.pdf]
